# Undergraduate dermatology education: The importance of curriculum review

**DOI:** 10.1002/ski2.288

**Published:** 2023-09-16

**Authors:** Nada Khalil, Laksha Bala, Christina George

**Affiliations:** ^1^ Department of Dermatology Charing Cross Hospital Imperial College Healthcare NHS Trust London UK; ^2^ Imperial College School of Medicine Imperial College London London UK

## Abstract

In this letter, we highlight the considerable diversity in undergraduate dermatology training in the United Kingdom and acknowledge the barriers faced in implementing the revised national undergraduate curriculum provided by the British Association of Dermatologists (BAD). We provide a pragmatic approach of ensuring that our dermatology placement aligns with the BAD national undergraduate curriculum and Medical Licensing Assessment (MLA) content map, in the face of limited clinician time and placement length. We urge other medical schools to adopt our approach of curriculum mapping, particularly in light of the upcoming MLA, and sustainable educational resource development.

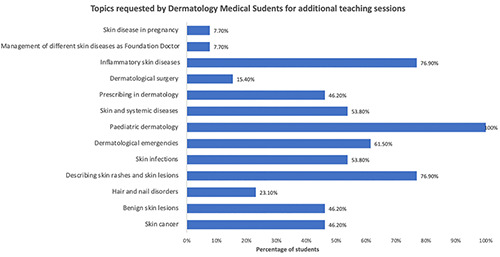

## Dear Editor,

There is considerable diversity in undergraduate dermatology training in the United Kingdom (UK), as well as limited opportunities for acquiring knowledge, beyond pursuing the specialty itself. There is an urgent need to improve dermatology teaching standards in UK medical schools. Inadequate training may lead to detrimental consequences, including delayed identification of skin cancers and mismanagement of serious dermatological conditions. Skin issues are among the most prevalent presenting complaints to general practice^1^ with around one in three graduating doctors^2^ going on to become general practitioners. Hence, the importance of providing high‐quality undergraduate dermatology education cannot be overstated.

There are significant discrepancies in the length and format of dermatology placements in UK medical schools. The majority offer 2‐week placements and very few provide dedicated teaching sessions during these rotations,^3^ highlighting the need for more structured and standardised teaching. In line with this, the General Medical Council is introducing a new standardised national undergraduate assessment from 2024; the Medical Licensing Assessment (MLA).^4^ Dermatology is included in the MLA content map, necessitating UK medical schools to modify their curriculum appropriately. To facilitate this transition, the British Association of Dermatologists (BAD) has recently revised its recommendations for the national undergraduate curriculum and aligned it with the MLA content map.^4^ However, implementing an updated dermatology curriculum is a complex and time‐consuming task. Clinicians face various obstacles, including time constraints, lack of training in mapping exercises, limited funding and inadequate infrastructure.^3^

Considering these barriers, we have developed a pragmatic and efficacious approach to ensure that our 2‐week dermatology placement at Imperial College London aligns with the BAD national undergraduate curriculum and MLA content map, whilst catering to the specific learning needs of our students. We now conduct a pre‐placement survey before each rotation (Figure 1a), allowing us to better understand students' learning needs and tailor their placement accordingly. This has resulted in a broader coverage of the dermatology curriculum, addressing areas that students felt were lacking in their previous clinical experience. Thirteen medical students completed our pre‐placement survey (Figure 1b). Students requested additional teaching in paediatric dermatology (100%), as well as inflammatory skin disease (76.9%), description of skin rashes and lesions (76.9%) and dermatological emergencies (61.5%). The extra teaching sessions provided in these areas achieved 100% student attendance. Post‐session surveys revealed that all students ‘strongly agreed’ that the sessions were useful for their future practice. Specifically, students expressed improved confidence in understanding dermatological terminology (100%), accurately describing skin rashes (92.3%), recognising and managing emergency dermatological presentations (84.6%), and identifying paediatric manifestations of common dermatological conditions (84.6%).

FIGURE 1(a) Pre‐placement dermatology survey. (b) Pre‐placement survey results.

**Figure:**
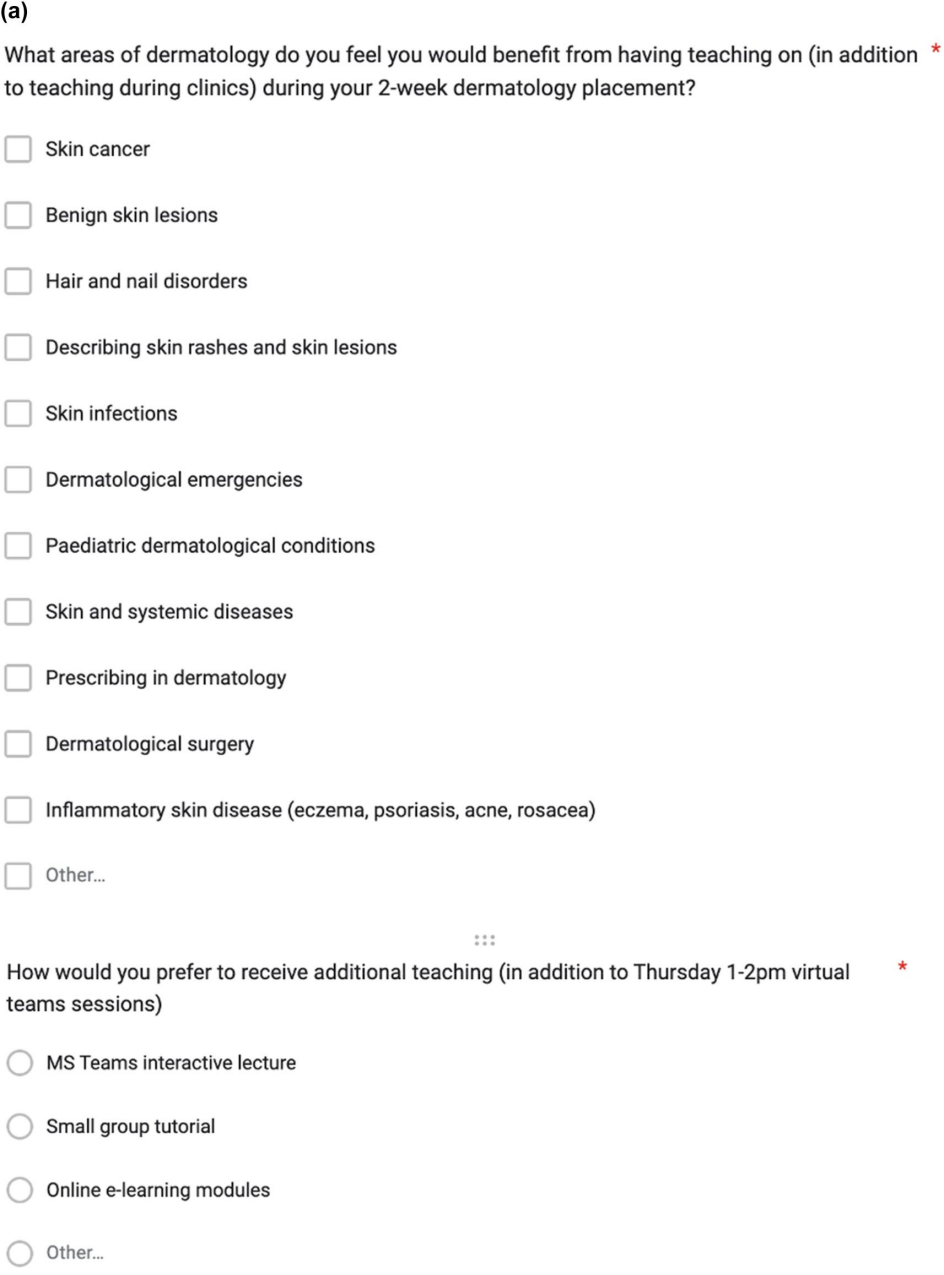

**Figure:**
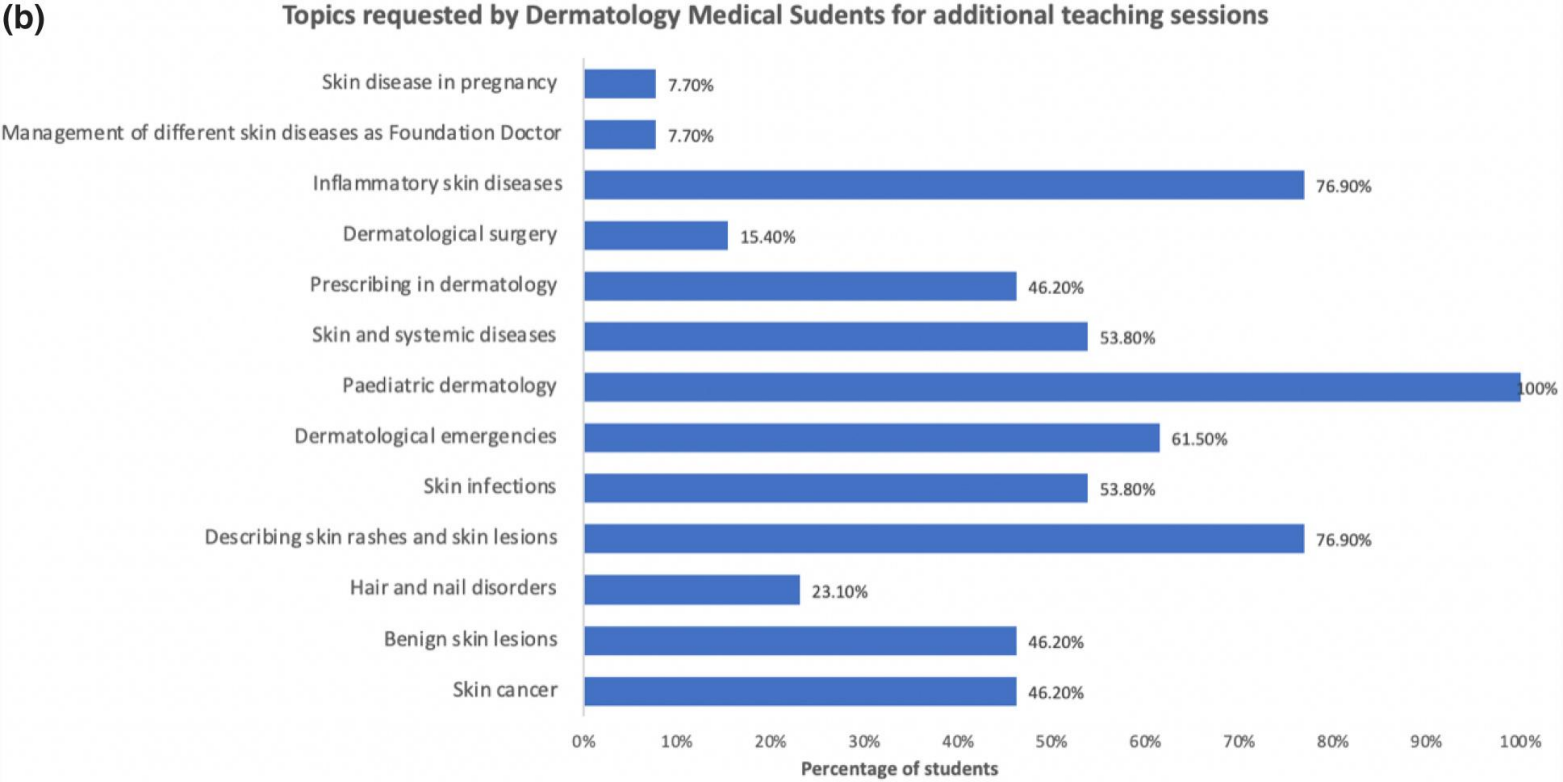

We acknowledge that the provision of additional teaching requires investment of clinicians' time, which may not be possible alongside clinical commitments. In view of this, we have also created a library of learning resources for our students, including pre‐recorded lectures on dermatological emergencies and skin cancer, as well as e‐learning modules covering inflammatory skin conditions. These resources will be accessible in subsequent academic years, ensuring sustainable availability of high‐quality educational content to support students' learning in dermatology. In addition, we also direct our students to resources created by the British College of Dermatology, the educational arm of the BAD, which has a dedicated Undergraduate Workstream providing resources for students and support for medical schools on implementing the undergraduate dermatology curriculum.^5^

The demand for additional training in Dermatology is also of great relevance to postgraduate trainees in non‐Dermatology specialities, as evidenced by over 250 survey responses we received from foundation, GP, emergency medicine and internal medical trainees in London requesting additional dermatology teaching. As a result, we created a monthly virtual Regional Dermatology Teaching Programme for postgraduates from all specialties, the impact of which we will be evaluating in the near future.

Improving provision of dermatology education in UK medical schools is crucial to equip future doctors with the knowledge and skills required to manage skin disease. We urge other medical schools to adopt our approach of curriculum mapping, particularly in light of the upcoming MLA, and sustainable educational resource development to ensure we are meeting the specific learning needs of our students.

## CONFLICT OF INTEREST STATEMENT

None to declare.

## AUTHOR CONTRIBUTIONS

**Nada Khalil**: Conceptualization (lead); data curation (lead); formal analysis (lead); investigation (lead); methodology (lead); project administration (lead); resources (lead); software (lead); validation (lead); visualization (lead); writing—original draft (lead); writing—review & editing (lead). **Laksha Bala**: Conceptualization (lead); data curation (lead); formal analysis (lead); investigation (lead); methodology (lead); project administration (lead); resources (lead); software (lead); validation (lead); visualization (lead); writing—original draft (lead); writing—review & editing (lead). **Christina George**: Supervision (lead); writing—original draft (supporting); writing—review & editing (supporting).

## FUNDING INFORMATION

This research received no specific grant from any funding agency in the public, commercial, or not‐for‐profit sectors.

## ETHICS STATEMENT

Not applicable.

## Data Availability

The authors confirm that the data supporting the findings of this study are available within the article.
